# "Wasting" Money in Public Health

**Published:** 1921-01-15

**Authors:** 


### "Wasting" Money in Public Health.

That excellent little publication, the monthly magazine of the Loyal Order of Ancient Shepherds, recalls in its current issue the fact that approximately 40,000 men were discharged from the Army suffering from tuberculosis in many forms, and that, with the influx of female labour during the war to the industrial army, there has been an increase of mortality from tuberculosis among women. The writer properly names as one of the factors governing this increase the lower nutritious food values which constitute one of our war legacies. The general resisting-power of the individual has been lowered, and this has had an effect—probably only temporary—on working women in this country, though the consequences are nothing like so marked as in certain European countries, especially Austria, where the tuberculosis mortality among women has risen from 2,633 in 1914 to 5,018 in 1919. The object of the writer in the *Shepherds' Magazine* is to call attention to the fact that many local authorities are receiving bitter complaints from ratepayers of the "waste of public money" in providing preventive treatment of tuberculosis. But this type of ignorant criticism local authorities can well afford to disregad. The time has passed when this or any other civilised country can afford to look upon the maintenance of public health as "a waste of public money," and, happily, the sense of the community in Great Britain is learning rapidly to realise that there is no truer national economy than a national effort to banish disease.

